# Cognitive impairment induced by sevoflurane anesthesia is mediated by the cholinergic system after gastrointestinal surgery in older patients: A randomized, controlled trial

**DOI:** 10.1002/ibra.12079

**Published:** 2022-11-17

**Authors:** Xing‐Xing Liu, Qing‐Xu Yang, Yi Guo, Miao He, Zhen‐He Yu, Qi Tian, Zhao‐Qiong Zhu

**Affiliations:** ^1^ Department of Anesthesiology Affiliated Hospital of Zunyi Medical University Zunyi Guizhou P.R. China; ^2^ Department of Gastrointestinal Surgery Affiliated Hospital of Zunyi Medical University Zunyi Guizhou P.R. China; ^3^ Department of Hepatobiliary and Pancreatic Surgery Affiliated Hospital of Zunyi Medical University Zunyi Guizhou P.R. China; ^4^ Department of Anesthesiology Clinical Medical College & Affiliated Hospital of Chengdu University Chengdu Sichuan P.R. China; ^5^ Baylor St Luke's Hospital Houston Medical Center Sugarland Texas USA

**Keywords:** central cholinergic system, delayed neurocognitive recovery, gastrointestinal surgery, older patients, sevoflurane anesthesia

## Abstract

Delayed neurocognitive recovery after surgery is associated with increased morbidity and mortality. However, its mechanism of action remains controversial and complex. A prospective, double‐blind, randomized controlled trial was performed at the Affiliated Hospital of Zunyi Medical University. Older patients (aged 65 years and older) who underwent gastrointestinal surgery were randomly divided into sevoflurane‐based or propofol‐based anesthesia groups. The Mini‐Mental State Examination was performed to evaluate cognitive function. Peripheral venous blood was collected to test the levels of choline acetyltransferase and acetylcholinesterase. A total of 75 patients were enrolled and 30 patients in each group completed the study. On Day 1 postoperation, patients in the sevoflurane group showed worse performance on the Mini‐Mental State Examination than patients in the propofol group. Lower blood choline acetyltransferase concentrations and higher acetylcholinesterase concentrations were observed in patients who had sevoflurane anesthesia than in patients who had propofol anesthesia 1 day postoperative. At 3 days postoperation, patients with sevoflurane‐ or propofol‐based general anesthesia did not differ regardless of Mini‐Mental State Examination score or choline acetyltransferase and acetylcholinesterase levels. Sevoflurane‐based anesthesia has short‐term delayed neurocognitive recovery in older surgical patients, which may be related to central cholinergic system degeneration.

## Graphical abstract

The purpose of this article was to explore the hypothesis that older patients receiving sevoflurane‐based anesthesia during gastrointestinal surgery have negative postoperative cognitive effects and that the central cholinergic system is involved. A prospective, double‐blind, randomized controlled trial was performed at the Affiliated Hospital of Zunyi Medical University. Older patients who underwent gastrointestinal surgery were randomly divided into sevoflurane‐based or propofol‐based anesthesia groups. The study found on Day 1 postoperation, patients in the sevoflurane group showed worse performance on the Mini‐Mental State Examination than patients in the propofol group. Lower blood choline acetyltransferase concentrations and higher acetylcholinesterase concentrations were observed in patients who had sevoflurane anesthesia than in patients who had propofol anesthesia 1 day postoperative. At 3 days postoperation, patients with sevoflurane‐ or propofol‐based general anesthesia did not differ regardless of Mini‐Mental State Examination score or choline acetyltransferase and acetylcholinesterase levels. This study demonstrated that sevoflurane‐based anesthesia has short‐term delayed neurocognitive recovery in older surgical patients, which may be related to central cholinergic system degeneration.

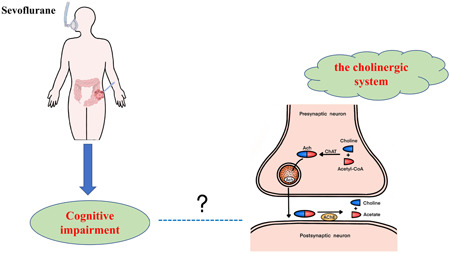

## 1 BACKGROUND

In China, more than 60 million patients undergo surgery annually.^1^ Postoperative cognitive dysfunction is a common postoperative complication that affects the central nervous system and is associated with increased medical costs, impaired patient recovery, and even increased mortality, especially in older patients.^2^, ^3^, ^4^, ^5^, ^6^, ^7^, ^8^ Deficits in cognitive functions, including learning and memory, are major clinical features of postoperative cognitive dysfunction.^9^ Recently, the term “delayed neurocognitive recovery” was adopted for cognitive impairment in the interval from 0 to 30 days postoperatively.^10^

With the rapidly increasing need for surgical procedures and an increase in the number of older patients undergoing elective surgical procedures that necessitate the use of anesthetic medications, accumulating studies have explored whether exposure to anesthesia might cause cognitive dysfunction, and whether a causative link to general anesthesia is increasingly recognized. Sevoflurane, an inhalation anesthetic, is widely used in general anesthesia owing to its advantages of rapid induction, rapid recovery, and stable anesthesia. The possible role of sevoflurane in cognitive impairment has been proposed based on findings from animal studies.^11^, ^12^, ^13^, ^14^ However, the role of sevoflurane in cognitive impairment in humans is limited. Although some experiments have shown that sevoflurane caused apoptosis and pathological changes in the rat hippocampus, leading to neurocognitive decline, whether sevoflurane contributes to the development of delayed neurocognitive recovery in the clinical setting, and the mechanism for this process in humans remains elusive. The effects of sevoflurane‐based anesthesia on outcomes in older patients remain to be elucidated.

**Figure 1:**
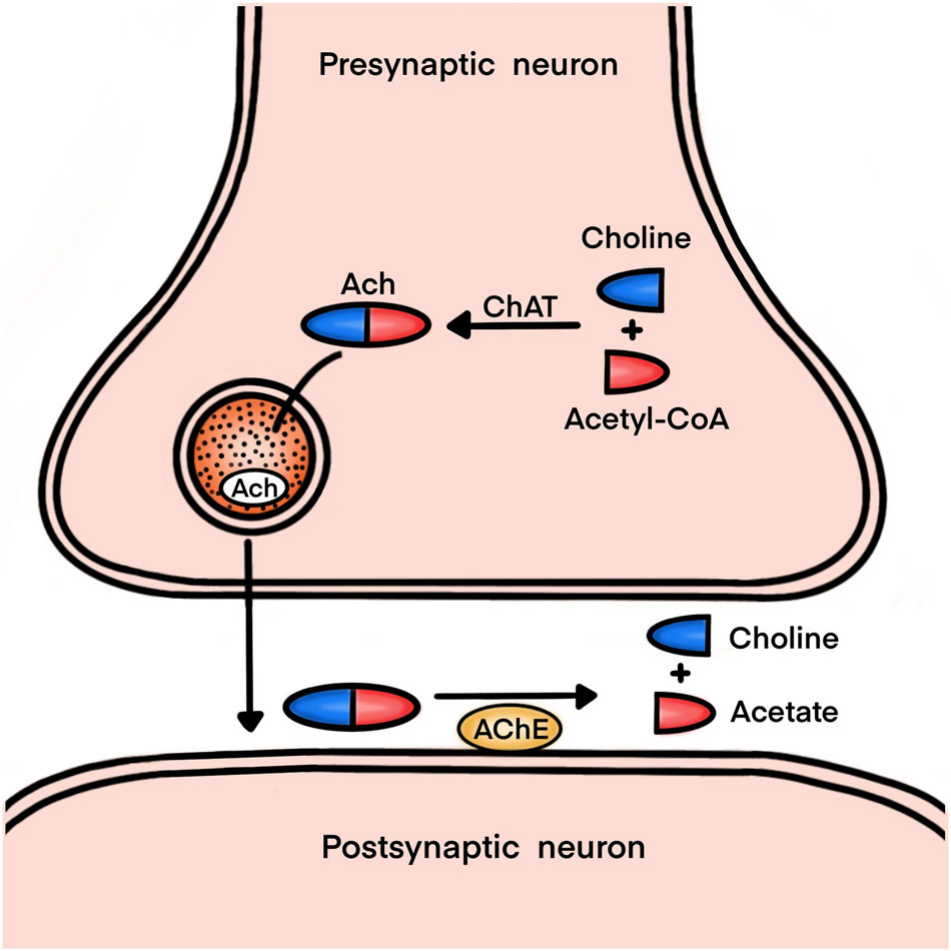
Synthesis and release of acetylcholine. Ach, acetylcholine; ChAT, choline acetyltransferase; Acetyl‐CoA, acetyl coenzyme A; AChE, acetylcholinesterase. [Color figure can be viewed at wileyonlinelibrary.com]

Accumulating evidence has demonstrated that the central cholinergic system is important in learning and memory.^15^, ^16^, ^17^, ^18^ Deficits in cholinergic transmission can potentially influence all aspects of cognition and behavior. Acetylcholine (ACh), which is involved in memory, was the first neurotransmitter to be identified.^19^, ^20^, ^21^ It is synthesized from choline and acetyl‐coenzyme A, catalyzed by choline acetyltransferase (ChAT). When cholinergic neurons are depolarized, ACh is released into the synaptic cleft, where ACh is rapidly inactivated by acetylcholinesterase (AChE), releasing choline and acetate (Figure 1). Therefore, we hypothesized that the central cholinergic system mediates the delayed neurocognitive recovery induced by sevoflurane in older patients undergoing gastrointestinal (GI) surgery.

The objective of this study was to examine the hypothesis that older patients receiving sevoflurane‐based anesthesia during GI surgery have negative postoperative cognitive effects and that the central cholinergic system is involved.

## 2 MATERIALS AND METHODS

### 2.1 Trial design

The present prospective, unicentric, double‐blind, randomized controlled trial was conducted from May 2018 to April 2019 at the Department of Anesthesiology, Affiliated Hospital of Zunyi Medical University. This study was conducted in accordance with the ethical standards of the 1964 Declaration of Helsinki and its later amendments. The study protocol was approved by the Biomedical Research Ethics Committee of the Affiliated Hospital of Zunyi Medical University (approval NO. 2018‐21). Each patient provided informed consent before surgery. We registered this work with the Chinese Clinical Trial Registry at http://www.chictr.org.cn (April24, 2018; ChiCTR1800015806) before patient enrollment.

### 2.2 Study participants

This study recruited older patients (≥65 years old) with physical status I‐II based on the American Society of Anesthesiologists (ASA) who were scheduled for elective GI surgery. The exclusion criteria included the following: refusal to participate in the study, central nervous system‐related disease, length of education of fewer than 6 years, preoperative Mini‐Mental State Examination (MMSE) score <24, current use of sedatives or antidepressants, history of allergy to inhaled anesthetics, canceled surgery or conversion to open surgery, and low compliance or inability to complete follow‐up visits.

### 2.3 Randomization and blinding

Enrolled patients were randomized to the sevoflurane and propofol‐based general anesthesia groups at a 1:1 ratio by a physician using a random number table. Each care provider, patient, and anesthetist responsible for assessments before and after surgery were blinded to the allocation of assignments throughout the entire process before completing the follow‐up examinations and conducting our final analyses.

### 2.4 Anesthesia and perioperative management

All subjects received general anesthesia and inhaled oxygen via masks after entering the operating room; electrocardiogram, heart rate, blood pressure (BP), and blood oxygen saturation were monitored. In accordance with the study protocol, 0.3 μg·kg^−1^ sufentanil, 0.2–0.3 mg·kg^−1^ etomidate, and 0.9 mg·kg^−1^ rocuronium were administered to each subject to induce general anesthesia. For anesthesia maintenance, sevoflurane inhalation (1.0–1.5 minimal alveolar content) combined with intravenous infusion of remifentanil (0.1–0.2 μg·kg^−1^·min^−1^) were administered to subjects in the sevoflurane group, and intravenous propofol (4–12 mg·kg^−1^·h^−1^) and remifentanil (0.1–0.2 μg·kg^−1^·min^−1^) infusions were administered to subjects in the propofol‐based group. Intraoperatively, the depth of anesthesia was monitored according to the bispectral index, which was maintained at 40–60 by controlling the sevoflurane or propofol dose. We maintained BP within ±20% of the baseline value and nasopharyngeal temperature at 36–37.5°C. Meanwhile, we controlled the tidal volume (6–8 ml/kg) to maintain the end‐tidal pressure of carbon dioxide level at 35–45 mmHg. After surgery, we transferred the subjects to the post‐anesthesia care unit for recovery. Within the first 48 h postoperatively, patient‐controlled analgesia was prescribed for each patient.

### 2.5 Variables

Preoperative evaluations were performed the day before surgery. Patient demographic data (such as sex, age, body weight, height, body mass index, and education level) and perioperative characteristics including ASA physical status, MMSE score, anesthesia and surgery duration, fluid volume given intraoperatively, intraoperative blood loss volume, and postoperative hospitalization days were recorded.

Cognitive function was evaluated using the MMSE score before surgery and at 1 day and 3 days postoperation. We obtained 3 ml venous blood pre‐anesthesia, 1 day postoperation, and 3 days postoperation for measuring ChAT and AChE, which may be related to delayed neurocognitive recovery.

### 2.6 Enzyme‐linked immunosorbent assay

After standing at room temperature (22–25°C) for a 30‐min period, we centrifuged the blood samples (4000*g*, 4°C) for a 20‐min period to obtain serum, which was later preserved at −80°C.^21^ We later determined the cholinergic biomarker levels, including ChAT and AChE, using enzyme‐linked immunosorbent assay (ELISA) kits to analyze the changes to the cholinergic nervous system following surgery.

### 2.7 Statistical analysis

SPSS V.23.0 (IBM Corp.) was employed for all statistical analyses.

### 2.8 Sample size

We determined the sample size according to the preliminary experimental results, and the MMSE score 1 day after surgery was taken as the primary outcome. During the pre‐experiment (19 patients were included in the pre‐experiment: 9 in the sevoflurane group and 10 in the propofol group), we observed a mean MMSE score at 1‐day postoperation of 25.12 ± 1.71 in the propofol group and 23.90 ± 1.65 in the sevoflurane group. To achieve 80% power and a two‐tailed type I error rate of *α* = 0.05, at least 30 patients were assigned to every group. Considering the loss‐to‐follow‐up rate of 20%, 75 patients were recruited in this study.

### 2.9 Analysis

The primary endpoint in the present work was the MMSE score on Days 1 and 3 after surgery. The secondary endpoints included alterations in the serum cholinergic biomarker contents (ChAT and AChE). These data were analyzed using repeated‐measures analysis of variance combined with Bonferroni multiple comparison corrections.

Categorical data are presented as frequencies (percentages); Fisher's exact test or chi‐squared test was used for the analysis. Continuous variables are expressed as medians (25th, 75th percentiles) or means ± standard deviations. Later, the Mann–Whitney *U*‐test or independent *t*‐test was used for comparisons based on data distribution. *p* < 0.05 was considered statistically significant.

## 3 RESULTS

**Figure 2:**
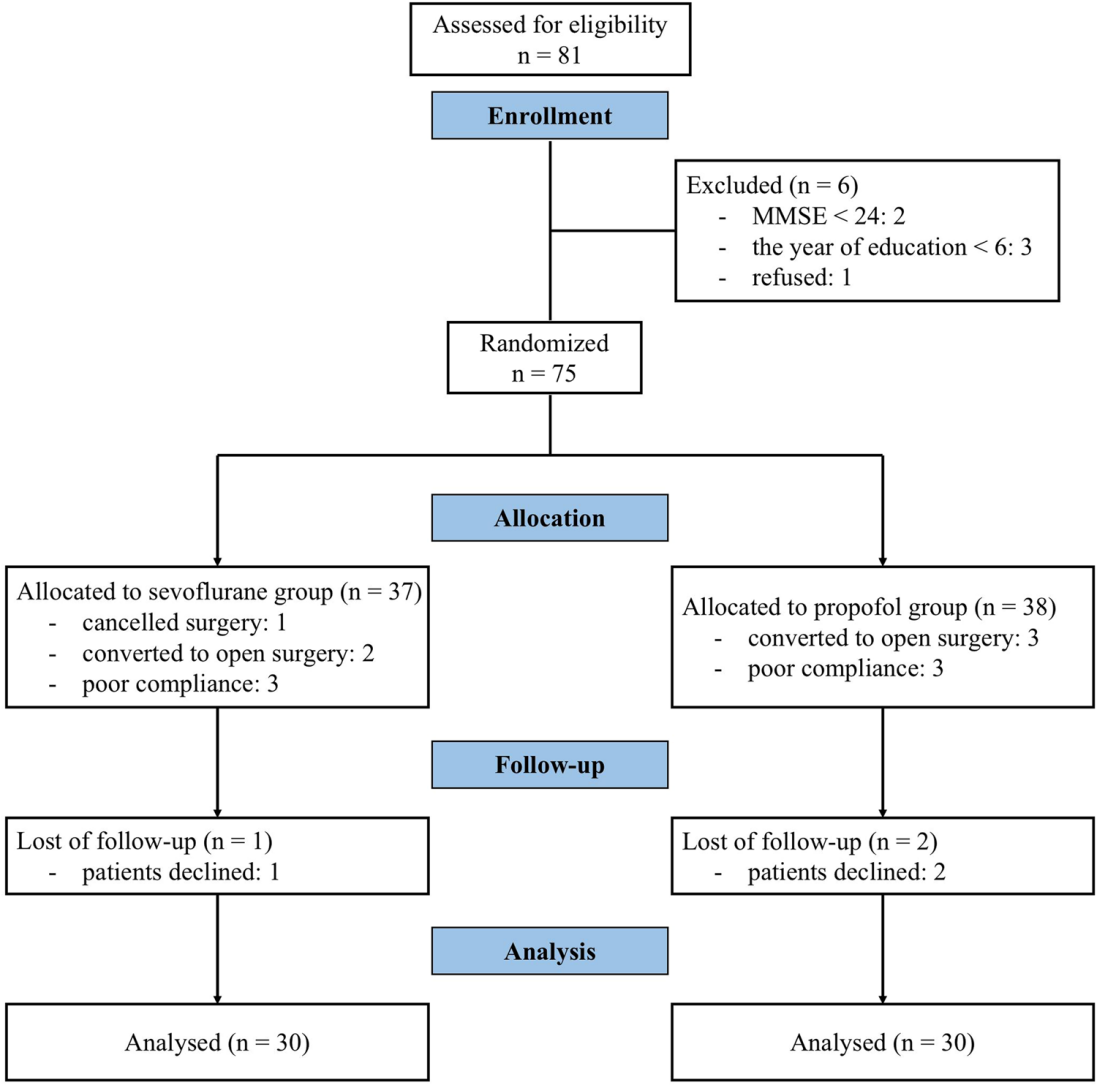
Flowchart of the study. MMSE, Mini‐Mental State Examination. [Color figure can be viewed at wileyonlinelibrary.com]

A total of 81 patients were screened for this study from May 2018 to April 2019. Among them, 6 patients did not meet the inclusion criteria, and, thus, 75 patients were enrolled. Among the enrolled patients, one patient canceled the surgery, five patients were converted to open surgery, six patients had poor compliance, and three patients declined. Therefore, 30 patients in each of the sevoflurane and propofol groups completed the study per protocol (Figure 2).

The baseline characteristics and perioperative variables of the patients are shown in Table 1. The average age was 69.0 (66.0–72.3) years in the propofol group and 69.0 (66.0–72.0) years in the sevoflurane group (*p* = 0.671). There were no significant differences in demographic and perioperative characteristics between the sevoflurane and propofol groups.

**Table 1:** Demographic and perioperative characteristics of the study participants

	Sevoflurane (*n* = 30)	Propofol (*n* = 30)	*p* Value*
Age (years)	69.0 (66.0,72.0)	69.0 (66.0, 72.3)	0.671
Sex			0.795
Female	14 (46.7)	13 (43.3)	
Male	16 (53.3)	17 (56.7)	
BMI (kg/m^2^)	22.33 ± 2.06	21.62 ± 2.92	0.283
Education (years)	7.0 (6.0, 9.0)	7.0 (6.0, 8.0)	0.333
ASA physical status			1.000
I	3 (10.0)	4 (13.3)	
II	27 (90.0)	26 (86.7)	
Operation time (min)	109.07 ± 36.90	97.20 ± 35.04	0.207
Anesthesia time (min)	151.30 ± 42.90	140.03 ± 41.24	0.304
Intraoperative infusion (ml)	1075.0 (900.0, 1300.0)	1050.0 (888.0, 1500.0)	0.994
Estimated blood loss (ml)	20.0 (10.0, 50.0)	20.0 (10.0, 50.0)	0.607
Postoperative hospitalization days	5.0 (3.8, 7.0)	6.5 (4.0, 8.3)	0.058

*Note*: The data are presented as the number of patients (*n*) and percentage (%), mean ± SD, or median (25th to 75th percentiles).

Abbreviations: ASA, American Society of Anesthesiologists; BMI, body mass index; SD, standard deviation.

* The *p*‐values were calculated by the independent sample *t*‐test, Mann–Whitney *U*‐test, *χ* ^2^ test, or Fisher exact test.

**Figure 3:**
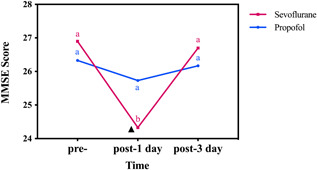
Comparison of MMSE scores between sevoflurane and propofol groups at different time points. a and b, the difference between time points indicated by the letter marking method (comparison within groups); ▲, there is a significant difference between the sevoflurane group and the propofol group (comparison between groups). MMSE, Mini‐Mental State Examination; pre‐, before anesthesia; post‐, operative. [Color figure can be viewed at wileyonlinelibrary.com]

Before surgery, there was no significant difference in the MMSE scores between the two groups (*p* > 0.05). At 1 day postoperation, patients in the sevoflurane group showed worse performance on the MMSE than those in the propofol group at the corresponding time point (*p* < 0.001). There were no significant differences in performance on the MMSE test at 3 days postoperation between patients in the sevoflurane and propofol groups (*p* = 0.183) (Table 2). Figure 3 shows the MMSE scores between the sevoflurane and propofol groups at different time points within and between groups.

**Table 2:** MMSE scores of the two groups at different time points

Group (*n* = 30)	MMSE score		
	Pre‐	Post‐ 1 day	Post‐ 3 day
Sevoflurane	26.90 ± 1.24	24.33 ± 1.24	26.70 ± 1.60
Propofol	26.33 ± 1.12	25.73 ± 1.57	26.17 ± 1.46
*p* Value	0.069	0.001	0.183

*Note*: Data are presented as the mean ± SD. Boldface values indicate *p* < 0.05.

Abbreviations: MMSE, Mini‐Mental State Examination; post‐, operative pre‐, before anesthesia; SD, standard deviation.

**Figure 4:**
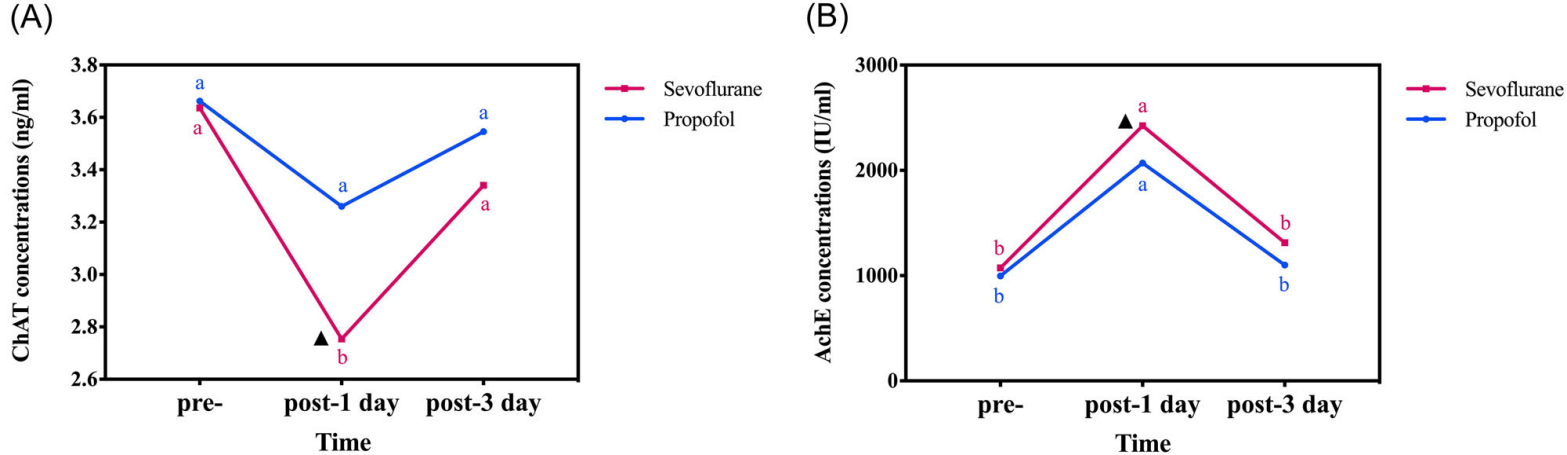
Comparison of ChAT (A) or AChE (B) between groups at different time points. a and b, the difference between time points indicated by letter marking method (comparison within‐group); ▲, there is a significant difference between the sevoflurane group and the propofol group (comparison between‐group). AChE, the enzyme acetylcholinesterase; ChAT, the enzyme choline acetyltransferase; pre‐, before anesthesia; post‐, operative. [Color figure can be viewed at wileyonlinelibrary.com]

ELISA analysis showed that ChAT concentrations were lower in patients who had undergone sevoflurane anesthesia than in patients who had undergone propofol anesthesia, with a significant difference at 1‐day postoperation (*p* = 0.007). AChE concentrations were higher in patients who had undergone sevoflurane anesthesia than in patients who had undergone propofol anesthesia, and were significantly different at 1‐day postoperation (*p* = 0.023). However, there was no difference in ChAT and AChE levels between the two groups at 3 days after surgery (Figure 4).

## 4 DISCUSSION

Sevoflurane is the most frequently used inhalational anesthetic. Delayed neurocognitive recovery is one of the most common cognitive disturbances occurring after surgery. Determining whether anesthetic choice plays a role in delayed neurocognitive recovery has long been the focus of research.^22^, ^23^, ^24^, ^25^, ^26^, ^27^ Although, anesthesia during surgery is thought to produce a neuroinflammatory state that leads to cognitive impairment, the choice of general anesthetics and their association with delayed neurocognitive recovery remains controversial. Determining the most appropriate anesthetic option is of great importance as anesthesia is a risk factor for delayed neurocognitive recovery. Our study showed that 1 day after GI surgery, patients in the sevoflurane group showed worse performance on the MMSE, lower blood ChAT concentrations, and higher AChE concentrations than patients who received propofol anesthesia. However, at 3 days postoperation, patients in the propofol‐based group did not differ from patients in the sevoflurane group with regard to MMSE score, ChAT concentration, or AChE concentration, suggesting that sevoflurane‐based anesthesia has short‐term negative postoperative cognitive effects in older surgical patients, which may be related to central cholinergic system degeneration.

There is a growing body of evidence from studies exploring the difference in postoperative cognitive dysfunction incidence between total intravenous‐ or inhalation‐based agents for general anesthesia. One trial in cardiac patients showed that compared with sevoflurane, propofol administration during cardiopulmonary bypass surgery helped to effectively decrease the incidence of delayed neurocognitive recovery without increased side effects.^28^ However, the incidence of propofol‐ and sevoflurane‐related postoperative cognitive dysfunction in noncardiac patients varies.^4^, ^22^, ^29^ Recently, a meta‐analysis including seven articles with a total of 869 patients was conducted and the results indicated that total intravenous anesthesia decreased postoperative cognitive dysfunction risk, with an odds ratio of 0.52 (0.31–0.87). However, the low certainty of the evidence may be ascribed to the use of different diagnostic approaches, varied assessment timing, and heterogeneities in the reported data.^30^ Debates among researchers continue within current studies as to whether the anesthetics alone are responsible for such short‐time actions on cognitive function. Another study reported that the incidence of postoperative cognitive dysfunction was similar after the use of propofol and sevoflurane for general anesthesia. Patients had measurable slowing of executive function and memory processing in the first days after anesthesia.^31^, ^32^ Our results also showed that 1 day after surgery, patients in the sevoflurane group showed worse performance on the MMSE.

The MMSE is a facile scale used to screen for cognitive impairment and is a commonly accepted method used to evaluate postoperative cognitive dysfunction.^33^, ^34^, ^35^ Although, it is reportedly insensitive to subtle changes in cognitive function, lower MMSE scores independently predicted the risk of cognitive impairment.^36^ As a result, MMSE scores were utilized for evaluating postoperative cognitive dysfunction in this study. Besides, declining working memory is one of the main concerns of postoperative cognitive dysfunction, and the central cholinergic system plays an important role in regulating cognitive function.^37^ Previous research has demonstrated that working memory dysfunction occurs in patients after cardiac surgery.^38^ It has been demonstrated that the cholinergic system plays a role in the learning process, and central cholinergic neuronal degeneration facilitates the development of postoperative cognitive dysfunction.^15^ Moreover, published data indicate that ACh is involved in memory.^17^ In adult mice, anesthesia/surgery impairs working memory, and the central cholinergic system is involved.^16^ However, results obtained from animal studies cannot be applied directly to clinical settings. In our study, we included patients aged 65 years or older who were scheduled to undergo elective GI surgery. Our results suggested that sevoflurane‐based anesthesia has short‐term delayed neurocognitive recovery in older surgical patients, which may be related to central cholinergic system degeneration. From a clinical perspective, this result may guide us that more attention should be paid to cognitive decline in elderly patients in the early postoperative period, but whether short‐term changes in ChAT, AChE, and MMSE scores change clinical practice may require further in‐depth studies.

Certain limitations should be considered in the present work. Firstly, the clinical trial was conducted at one center rather than at multiple sites. A multicenter and well‐controlled randomized controlled trial is warranted to determine whether particular anesthetics have more favorable risk profiles. Second, the correlation between ChAT, AChE, and MMSE score was not further performed; therefore, we do not know whether there is an association between the three. Third, postoperative follow‐up was performed over 3 days; therefore, data on the long‐term (e.g., 10, 20, 30, or even longer) postoperative effects of sevoflurane‐based anesthesia on older patients are not available.

## 5 CONCLUSIONS

This study demonstrated that sevoflurane‐based anesthesia has short‐term delayed neurocognitive recovery effects in older patients who have undergone GI surgery, which may be related to central cholinergic system degeneration.

## AUTHOR CONTRIBUTIONS

*Study design, drafting the article, critical revision of the article*: Xing‐Xing Liu. *Collected the data and interpretation*: Qing‐Xu Yang, Yi Guo. *Analyzed the data*: Miao He. *Performed the ELISA*: Zhen‐He Yu. *Modify the manuscript*: Qi Tian. *Study design, controlled the research quality, and critically revised the manuscript*: Zhao‐Qiong Zhu. All authors have read and approved the manuscript.

## CONFLICT OF INTEREST

The authors declare no conflict of interest.

## ETHICS STATEMENT

This study was performed in accordance with the Declaration of Helsinki, and the trial protocol was approved by the Biomedical Research Ethics Committee of the Affiliated Hospital of Zunyi Medical University (approval NO. 2018‐21). Written informed consent was obtained from all participants before surgery.

## Data Availability

The authors confirm that the data supporting the findings of this study are available within the article and its supplementary materials.
